# Preservation of Health

**Published:** 1846-03

**Authors:** 


*Preservation of Health.* By Jno. C. Warren, M. D., Professor of Anatomy and Surgery in Harvard University. Boston: William D. Ticknor & Co., 1846, pp. 90.

This little work was originally a lecture before the American Institute, since which additions have been made, and the whole published. It contains much valuable practical instruction, and would be well to be in every family, and carefully read; and the most of its precepts, if practised, would secure, in a great measure, the health of the individual, and immunity from disease.

